# The dependency of fetal left ventricular biomechanics function on myocardium helix angle configuration

**DOI:** 10.1007/s10237-022-01669-z

**Published:** 2022-12-22

**Authors:** Laura Green, Wei Xuan Chan, Meifeng Ren, Citra Nurfarah Zaini Mattar, Lik Chuan Lee, Choon Hwai Yap

**Affiliations:** 1 Department of Bioengineering, Imperial College London, London, UK; 2 BHF Centre of Research Excellence, Imperial College London, London, UK; 3 Department of Biomedical Engineering, National University of Singapore, Singapore, Singapore; 4 Department of Obstetrics and Gynecology, National University Health Systems, Singapore, Singapore; 5 Yong Loo Lin School of Medicine, National University of Singapore, Singapore, Singapore; 6 Department of Mechanical Engineering, Michigan State University, East Lansing, USA

**Keywords:** Fiber orientation, Fetal cardiac function, Fetal heart biomechanics, Myocardial strain, Finite element model

## Abstract

The helix angle configuration of the myocardium is understood to contribute to the heart function, as finite element (FE) modeling of postnatal hearts showed that altered configurations affected cardiac function and biomechanics. However, similar investigations have not been done on the fetal heart. To address this, we performed image-based FE simulations of fetal left ventricles (LV) over a range of helix angle configurations, assuming a linear variation of helix angles from epicardium to endocardium. Results showed that helix angles have substantial influence on peak myofiber stress, cardiac stroke work, myocardial deformational burden, and spatial variability of myocardial strain. A good match between LV myocardial strains from FE simulations to those measured from 4D fetal echo images could only be obtained if the transmural variation of helix angle was generally between 110 and 130°, suggesting that this was the physiological range. Experimentally discovered helix angle configurations from the literature were found to produce high peak myofiber stress, high cardiac stroke work, and a low myocardial deformational burden, but did not coincide with configurations that would optimize these characteristics. This may suggest that the fetal development of myocyte orientations depends concurrently on several factors rather than a single factor. We further found that the shape, rather than the size of the LV, determined the manner at which helix angles influenced these characteristics, as this influence changed significantly when the LV shape was varied, but not when a heart was scaled from fetal to adult size while retaining the same shape. This may suggest that biomechanical optimality would be affected during diseases that altered the geometric shape of the LV.

**Supplementary Information:**

The online version contains supplementary material available at 10.1007/s10237-022-01669-z.

## Introduction

The heart is one of the first organs to develop in gestation and undergoes highly dynamic growth and remodeling before birth (Tan and Lewandowski 2020). Cardiac development begins with the formation of cardiac tubes, which pulsate by 4 weeks gestation (WG). By 8–9 WG, the fetal heart progresses to the four-chamber configuration (Dhanantwari et al. 2009). At this time, the myocardium is roughly isotropic and homogeneous, with no coordinated alignment of the cardiomyocytes, but between 14 and 19 WG, it remodels into an anisotropic one, where cardiomyocyte alignments are similar to the adult heart (Mekkaoui et al. 2013). From then till birth, the extent of tissue anisotropy continues to increase (Mekkaoui et al. 2013). However, the mechanism for this remodeling and stimuli required for it are poorly understood.

The adult left ventricular (LV) myocardium tissue architecture is characterized by a transmurally varying helix angle (Mekkaoui et al. 2012), where myocyte bundle orientations follow a helical structure with a right-handed orientation (positive helix angle) at the endocardium, a left-handed orientation (negative helix angle) at the epicardium, and a near circumferential orientation (zero-helix angle) at the mid-wall. The transmural helix angle variation is typically reported to be roughly linear (Mekkaoui et al. 2012), with an epicardial-to-endocardial transmural difference of about 100° (Rohmer et al. 2007). In neonatal and infant LVs, Yang et al. (2018) reported a similar finding, where the helix angle had an epicardial-to-endocardial variation of − 60° to + 60°.

This was similarly reported for fetal hearts. Ohayon et al. (1999) performed histological evaluation of 3 fetal hearts at 14, 20, and 33 WG and reported epicardial-to-endocardial helix angle variation of − 55° to + 55°. In more recent work, Nishitani et al. (2020) used diffusion tensor magnetic resonance imaging on 20 fetal specimens aged 8–24 WG to investigate transmural fiber orientation, reporting transmural variations that were roughly linear, with a transmural difference of between 80° and 120° across the specimens. In their 24 WG samples, epicardial-to-endocardial transmural variation was approximately − 50° to + 60° (Nishitani et al. 2020). Using X-ray phase-contrast imaging, Garcia-Canadilla et al. (2018) reported the helix angle of a normal 19 WG fetal LV to have an epicardial-to-endocardial transmural variation of approximately − 50° to + 100° that was roughly linear.

Past computational studies have investigated the effects of helix angle configurations on LV function and attempted to explain the biomechanical rationale for these observed helix angle configurations in adult hearts. Palit et al. (2015) demonstrated that the helix angle configuration affected the passive stiffness of the LV. Vendelin et al. (2002) showed that helix angle affected the ejection fraction, spatial variability of stress and strains, and the estimated efficiency of the LV and reported that the typical helix angle configuration coincided with the configuration with maximum efficiency and minimal spatial variability of strains. Rijcken et al. (1999) performed iterative optimization of the helix angle configuration that targeted the minimization of the spatial variability of strains and reported that the resulting helix angle configurations matched literature values, suggesting that naturally occurring helix angle configurations minimized strain spatial variability. Pluijmert et al. (2012) and Washio et al. (2020) simulated the adaptive remodeling of the local helix angles, where helix angles were iteratively revised to reduce cross-fiber shear strain (for Pluijmert et al.) or to point to the direction of the greatest active tension (for Washio et al.) and similarly reported that this resulted in a final helix angle configuration that was physiological. These results suggested that the biomechanical environment, such as spatial variability and directionality of stresses and strain, may be guiding the development of the helix angle configuration.

Currently, however, there are no data on how the LV helix angle affects cardiac biomechanics in the fetal LV, or whether the helix angle configurations reported in the literature coincide with biomechanical optimality. In the fetal heart, the LV has a slightly different geometry and contractility is lower than adult hearts, and as such, investigations on fetal hearts are necessary, rather than extrapolating from adult heart data. This is especially so when it is currently unclear how much the shape and size of the LV affect biomechanical characteristics and optimality. To address these issues, we performed a series of finite element (FE) simulations over a wide range of helix angles, including patient-specific and idealized LV geometries. Our investigations are also necessary to inform FE simulations of fetal heart biomechanics, which have in the past provided insight into fetal heart physiology and pathophysiology (Dewan et al. 2017; Ong et al. 2020). In such simulations, the helix angle configuration is an important input that cannot be obtained from clinical scans, and it is important to understand the implication of varied helix angle configurations.

## Methods

### Overall approach

In this manuscript, we report these following investigations to enhance our understanding of the relationship between helix angle configurations and fetal LV biomechanics.

1. We first performed FE simulations of a patient-specific fetal LV linked to a lumped parameter model and compared it to FE simulations with LV volume over time data prescribed from measurements from echo images, to show that these two approaches have similar results. This comparison was intended to provide confidence for our subsequent results.
2. We then performed volume-constrained FE simulations for LVs of a further 4 fetal subjects, to gain an understanding of the effects of helix angle on biomechanics outcomes, and to understand whether literature discovered fetal LV helix angle configurations were close to the helix angle configuration that achieved optimal (maximum or minimum) biomechanical outcomes.
3. Next, we performed a comparison of myocardial strains obtained from FE versus that obtained from image tracking, to understand what helix angle configurations allowed a good fit between these two data sets, to inform future FE modeling work.
4. Finally, using a series of idealized LV geometries, we performed volume-constrained FE simulations to understand how much LV shape and size will affect LV biomechanics and affect the relationship between helix angles and biomechanics outcomes. In this investigation, using idealized geometries enabled better control over the LV shape and size as opposed to using patient-specific geometries.

### Image acquisition

4D echocardiography images of 5 healthy fetuses, 3 at 22 WG and 2 at 32 WG, were used in this study. The images were acquired from the National University Hospital, Singapore, with approval from the Domain Specific Review Board under protocol 2014/00056 and informed consent from all participants. 4D echo acquisition was performed using the Spatio-Temporal Image Correlation (STIC) mode with the GE Voluson 730 ultrasound attached to the RAB 4-8L transducer (GE Healthcare Inc., Chicago, Illinois, USA), with an axial resolution of approximately 154 µm and lateral resolution of approximately 219 µm based upon a 5 MHz transducer. Images were exported as a stack of 3D images over time as previously described (Wiputra et al. 2020).

### LV model reconstruction and motion estimation

**Fig. 1:**
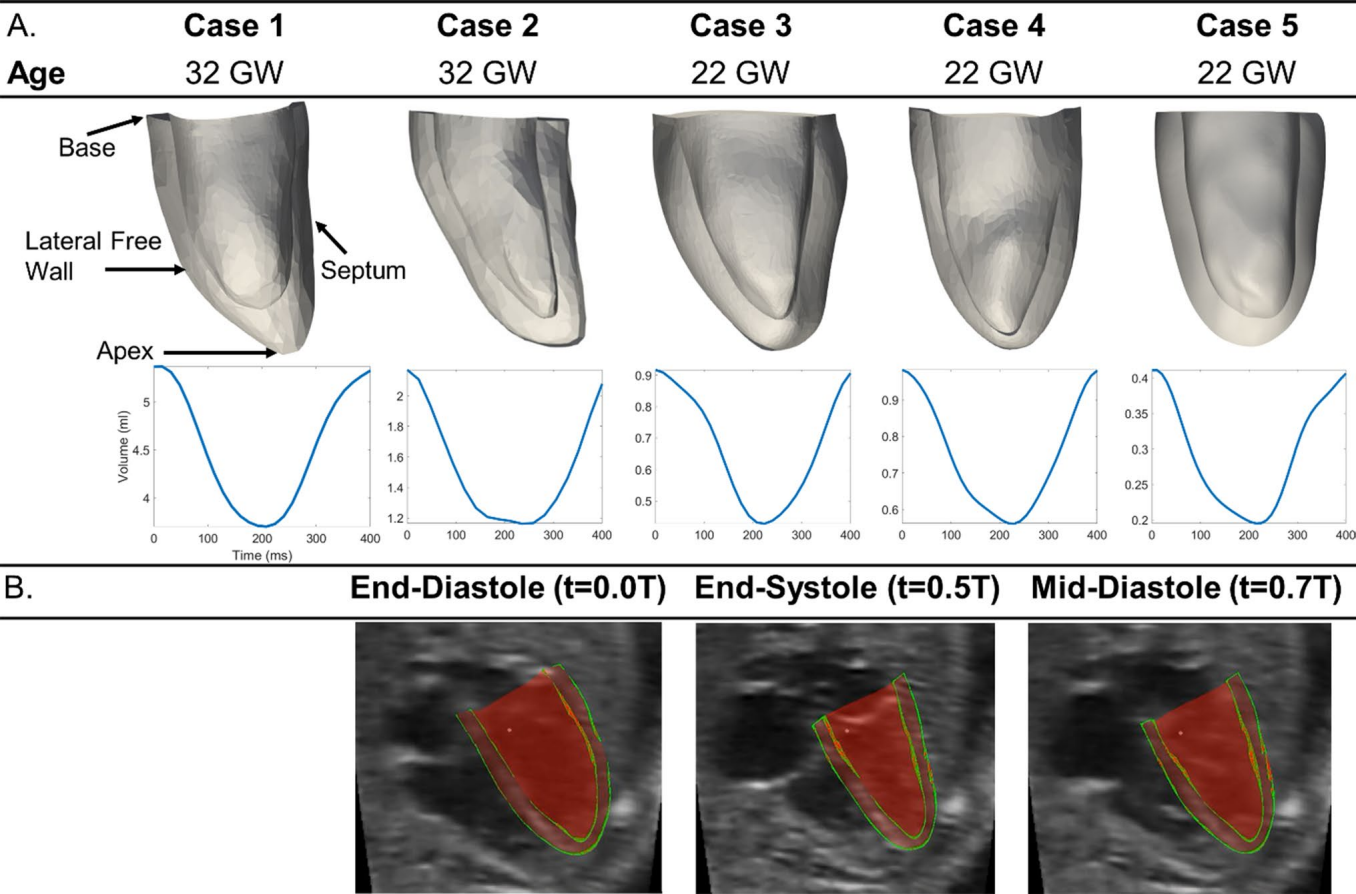
**A** Reconstructed patient-specific fetal LV geometries, their gestational ages, and volume–time curves. **B** 3D reconstructions of Case 1’s LV geometry superimposed onto a cross section of the echo image, at various time points in the cardiac cycle (t = 0 corresponds to end-diastole, while T is the duration of a cardiac cycle). Red—3D LV reconstruction, green—portions of the 3D reconstruction that was close to the image plane

Patient-specific geometries of the LV myocardium were segmented using a semi-automatic custom-written lazy-snapping algorithm (Wiputra et al. 2016). A validated, robust cardiac motion estimation algorithm was then used to track the motion of the LV myocardium (Wiputra et al. 2020), which is available at https://github.com/WeiXuanChan/motionSegmentation. Segmentation was only needed at one time point, and the reconstructed LV was propagated to all other time points with the calculated cardiac motion. Figure 1A shows the 5 patient-specific LV geometries and their volume–time curves. Figure 1B and supplementary video 1 show the motion tracking for fetal Case 1, demonstrating that motion tracking was robustly performed. Myocardial strains were computed from the estimated motions in the form of Green–Lagrange strains in the longitudinal and circumferential directions. Patient-specific geometries are available at https://figshare.com/articles/journal_contribution/Supplementary_Geometries/19487045.

### Lumped parameter model of the fetal circulation

**Fig. 2:**
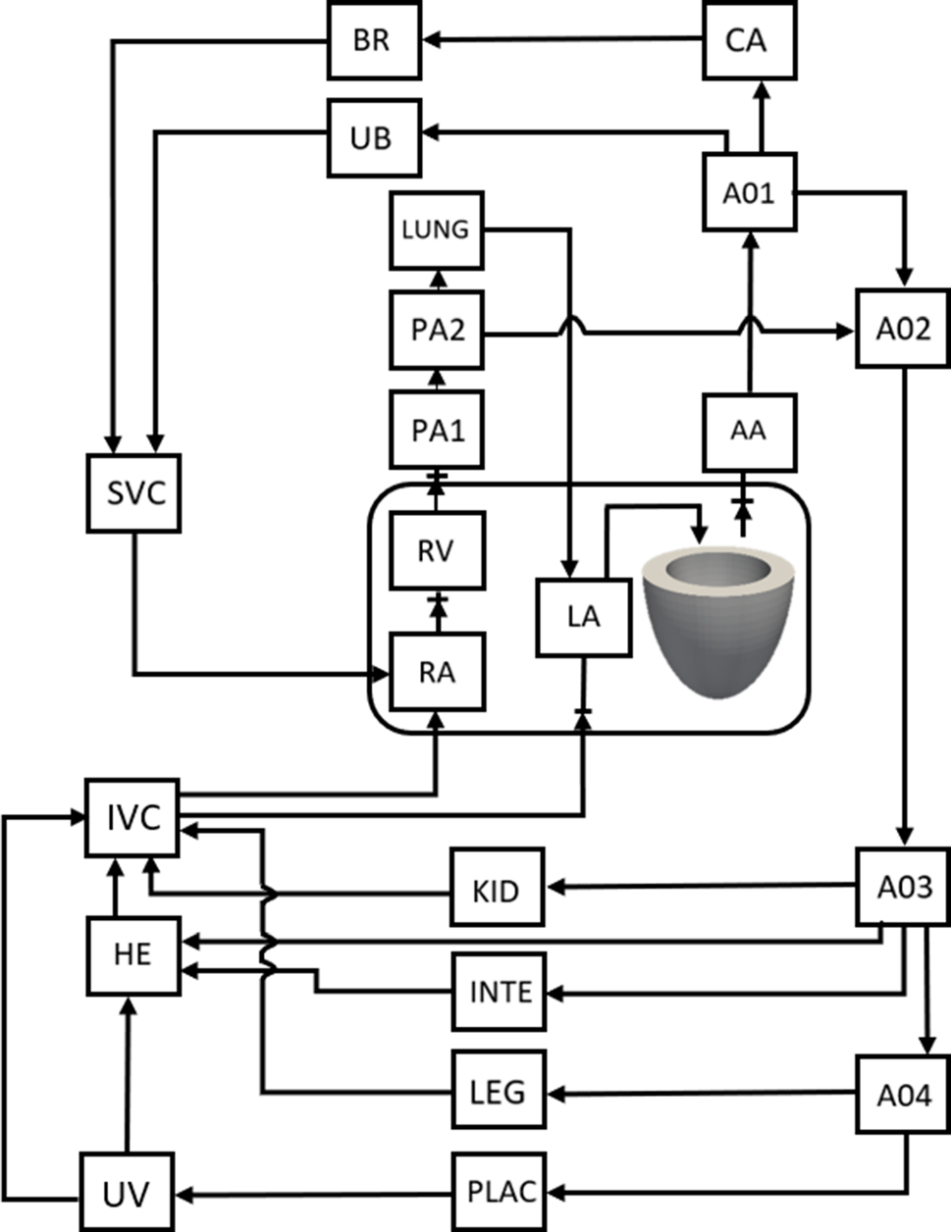
Schematic of the lumped parameter model used, adapted from Pennati et al. (1997), but recalibrated for our fetal LV case. Recalibration process and model parameter values are given in the supplementary text

A lumped parameter model was adopted to simulate the fetal circulation using electronic components as analogues for flow resistances and compliances, and it was coupled with the FE model to enable ventricular–vascular coupling. The lumped parameter model was coded with Ngspice (www.ngspice.sourceforge.net), an electrical circuit simulator. It is illustrated in Fig. 2, and parameter values are listed in Supplementary Table S2. The model was adopted from Pennati et al. (1997) with adjustments. Pennati et al.’s model was calibrated based upon full-term fetal human and lamb data (Pennati et al. 1997; Pennati and Fumero 2000), but we recalibrated it such that our FE and lumped parameter modeling for fetal Case 1 (32 WG) will match more recent invasive human fetal LV pressure measurements (Johnson et al. 2000), and descending aorta pulse pressure data (Versmold et al. 1981). The recalibration was achieved by scaling all resistances with a resistance scale factor, and all compliances with a compliance scale factor. Details of these adjustments are given in the supplementary text. This minor rescaling was necessary to account for the patient-specific features of fetal Case 1; for example, its stroke volume was higher than average, because Pennati et al.’s model describes the average fetus, not patient-specific cases. The lumped parameter model was executed for 12 cycles, to ensure that steady state is achieved.

### Finite element simulations of LV myocardial mechanics

Our FE methods were modified from previous work (Shavik et al. 2017; Ong et al. 2020) and were run using FEniCS (www.fenicsproject.org). The source code is available at https://github.com/WeiXuanChan/heartFEM. The myocardial helix angle was assumed to vary linearly from the epicardial boundary to the endocardial boundary for all locations on the LV wall, according to observations by past experimental studies (Nishitani et al. 2020). We define helix angle configuration by the transmural-averaged angle $(\overline{\tau })$ and the epicardial-to-endocardial transmural angle difference (${\tau }_{\mathrm{diff}}$), such that:

1 $$\begin{array}{*{20}l} {{\text{epicardium }}\;\;{\text{fiber}}\;\;{\text{ orientation}} = \overline{\tau } - \frac{1}{2}{\tau}_{{{\text{diff}}}} ,} \hfill \\ {{\text{endocardium}}\;\;{\text{ fiber}}\;\;{\text{ orientation}} = \overline{\tau} + \frac{1}{2}{\tau}_{{{\text{diff}}}} } \hfill \\ \end{array}$$

Based on literature reported fetal helix angle configurations $\overline{\tau }\cong 1{0}^{\circ} \mathrm{and} {\tau }_{\mathrm{diff}}\cong {123}^{\circ}$ are the average fetal helix angle configuration (Ohayon et al. 1999; Garcia-Canadilla et al. 2018; Nishitani et al. 2020). A range of configurations were tested, where $\stackrel{-}{\tau ,}{\tau }_{\mathrm{diff}}\in \left[-{90}^{\circ} , {180}^{\circ} \right]$. The myofiber transverse angle was assumed to be ${0}^{o}$ for the bulk of the simulations, as reasonable values of transverse angles were found not to affect our targeted results, as demonstrated in the supplementary material.

The passive stiffness of the myocardium was described with a transversely isotropic hyperelastic Fung-type formulation (strain energy function):

2a $$W= \frac{1}{2}C\left({e}^{Q}-1\right),$$

2b $$W=\frac{1}{2}C\left({e}^{{b}_{\mathrm{ff}}{E}_{\mathrm{ff}}^{2}+{b}_{\mathrm{xx}}\left({E}_{\mathrm{ss}}^{2}+{E}_{\mathrm{nn}}^{2}+{E}_{\mathrm{sn}}^{2}+{E}_{\mathrm{ns}}^{2}\right)+{b}_{\mathrm{fx}}\left({E}_{\mathrm{fn}}^{2}+{E}_{\mathrm{nf}}^{2}+{E}_{\mathrm{fs}}^{2}+{E}_{\mathrm{sf}}^{2}\right)}-1\right),$$

where *E* is the Green–Lagrange strain tensor with subscripts *f*, *s,* and *n* denoting myocardial fiber, sheet and sheet normal orientations. The stiffness parameters are defined by *b* and *C*. Myocardial stiffness was assumed to be the same as adult hearts, based on an analysis of available literature as reported earlier (Ong et al. 2020), and stiffness parameters are given in supplementary text Table S1.

Active stress $({P}_{\mathrm{act}})$ was calculated based on a published calcium activation model (Guccione et al. 1993; Fan et al. 2021; Shavik et al. 2021), which describes the sigmoidal relationship of chemical activation and tension of the cardiac muscle:

3 $${P}_{\mathrm{act}}={T}_{0\mathrm{LV}}\frac{{\mathrm{Ca}}_{0}^{2}}{{\mathrm{Ca}}_{0}^{2}+E{\mathrm{Ca}}_{50}^{2}}{C}_{t},$$

where ${T}_{0\mathrm{LV}}$ is the maximum tension. ${\mathrm{Ca}}_{0}^{2}$, ${\mathrm{ECa}}_{50}^{2},$ and ${C}_{t}$ describe the calcium activation behaviour, with ${\mathrm{C}a}_{0}$ representing the peak calcium concentration, ${\mathrm{EC}a}_{50}$ describing the calcium sensitivity dependent on sarcomere length, and ${C}_{t}$ describing the temporal variation (Guccione et al. 1993), described below:

4 $${C}_{t}=\frac{1}{2}(1-\mathrm{cos}\omega )$$

$\omega$ is dependent on the cycle time, with the variation:

5 $$\omega = \left\{ {\begin{array}{*{20}l} {\pi \frac{t}{{t_{0} }} } \hfill & {{\text{when}} \;0 \le t < t_{0} ,} \hfill \\ {\pi \frac{{t - t_{0} + t_{r} }}{{t_{r} }}} \hfill & { {\text{when}} \;t_{0} \le t < t_{0} + t_{r} } \hfill \\ 0 \hfill & { {\text{when}} \;t_{0} + t_{r} \le t,} \hfill \\ \end{array} } \right.$$

where ${t}_{0}$ is the time to peak tension, which specifies how long it takes the myofibers to contract, and ${t}_{r}$ is the relaxation time (Guccione et al. 1993) and is calculated using the following equation:

6 $$t_{r} = ml + b$$

where *m* is the gradient of linear relaxation duration with sarcomere length relation, *b* is the time intercept of linear relaxation duration with sarcomere length, and *l* is the sarcomere length, dependent on the degree of myocyte stretch.

Maximum tension ${T}_{0\mathrm{LV}}$ was reported to range from 23.9 to 59.2 kPa for fetal hearts (Racca et al. 2016). Here, it was iteratively adjusted such that peak systolic pressure in the simulation for a helical angle configuration of $(\overline{\tau }={0}^{\circ} , {\tau }_{\mathrm{diff}}={120}^{\circ} )$ matches measurements from Johnson et al. (2000). Time to peak tension ${t}_{0}$ was specified to be 140.5 ms for all fetal models, which was interpolated from Mulieri et al.’s (1992) measurements assuming a cardiac cycle duration of 400 ms, as all our fetal subjects had cardiac cycles duration close to this. The parameters *m* and *b* were scaled down from the values assumed in adult hearts simulations (Shavik et al. 2018), by assuming that they vary linearly with the cardiac cycle duration, but further minor adjustments were made to bring about more physiological pressure–volume loops. Parameter values are given in Table S1 in the supplementary material.

The zero-pressure unloaded state of the LV was calculated using a backward displacement method (Finsberg et al. 2018), based on a specified end-diastolic pressure and myocardium stiffness. Briefly, an initial guess of the pressure at the starting geometry was made, and this pressure value was iteratively adjusted by matching the end-diastolic pressure of the LV after pressure loading it to the end-diastolic volume. Subsequently, inverse displacement was applied to the starting geometry reducing LV pressure to zero to obtain the estimated unloaded state.

The LV models are meshed with a minimum of 2500 quadratic tetrahedral elements, in accordance with a previous mesh convergence study (Ong et al. 2020). The FE simulation was performed using FEniCS, minimizing the Lagrangian function detailed by Shavik et al. (2018), where boundary conditions were the same as previously reported, including a weak spring constant at the epicardium describing the effect of the tissue surrounding the heart, and a constraint at the base of the LV in the longitudinal direction but not in other directions. LV pressure was iteratively solved at each specified target volume obtained from the lumped model at each time step (Fan et al. 2021; Shavik et al. 2021). The resultant LV pressure and rate of volume change calculated from the FE simulation was fed back to the lumped parameter model at each time step.

### Volume-constrained finite element simulations

To investigate the LV of multiple subjects, multiple idealized LV geometries, and a wide range of helix angle configurations, we performed FE simulations at reduced computational cost for most LV geometries, by prescribing the LV volume over time waveform during the FE rather than connecting the FE to the lumped parameter model, using volume waveforms obtained from motion tracking of clinical echo images. The unloaded geometry was assumed to be the starting geometry at 1/3 diastolic duration, as the literature suggests that this is when the LV has the minimum pressure (Wenk et al. 2012; Gao et al. 2014; Di Achille et al. 2018). We could show that the results from this approach were sufficiently similar to those obtained from the FE with lumped parameters approach, when investigating the effects of helix angle on LV biomechanics.

### Finite element simulations of idealized LV geometries

**Fig. 3:**
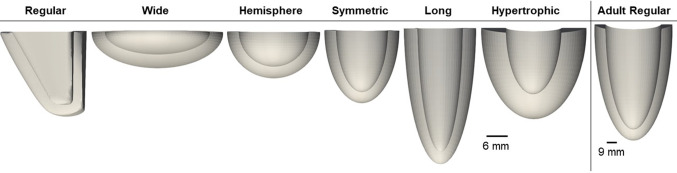
Idealized LV geometries of idealized and extreme dimensions, used to test how LV geometry affects the influence of LV helix angle on biomechanics function

To investigate how the LV geometry affects the influence of LV helix angle configuration on its biomechanics function, we generated 1 idealized asymmetric and 5 idealized symmetric prolate LV models with extreme dimensions, using SolidWorks CAD software, as shown in Fig. 3. These geometries, along with the patient-specific geometries, are available on https://figshare.com/articles/journal_contribution/Supplementary_Geometries/19487045. The “Regular” geometry was generated to be close to a physiologically normal fetal LV at 32 WG, with a straight septal wall, 3 mm wall thickness, 18.75 mm cavity length, and 15.40 mm basal cavity width (Daimei et al. 2014; Devore et al. 2019). The models evaluating alternative morphologies were based upon the “Symmetric” geometry, a half prolate ellipsoid, with a cavity length of 16.00 mm, and basal cavity width of 13.40 mm, with a 3 mm wall thickness. The “Hypertrophic” model was generated by doubling the wall thickness from the “Symmetric” geometry, the “Long” geometry was elongated to have cavity length that was 1.5 times that of the “Symmetric” geometry, and the “Hemisphere” geometry was modeled as a half sphere, while the “Wide” geometry was modeled as a half oblate ellipsoid. All these models had similar cavity volumes, within a 2% error, where end-diastolic volume (EDV) was 2.2 ml (Devore et al. 2019). The volume-constrained FE modeling was performed on these geometries, using FE parameters of human subject Case 2, and using the volume over time waveform measured from echo images of the same subject.

To test the effect of LV size on the relationship between LV helix angle and its biomechanics, we generated an idealized model of a human adult LV, assuming 9 mm wall thickness, 85 mm cavity length, and 45 mm basal cavity width (Gibson et al. 2014; Velagaleti et al. 2014) (Fig. 3). The volume waveform was adapted from the literature (Khalafvand et al. 2017) and scaled to an EDV of 132 ml and a stroke volume of 81.8 ml (Gibson et al. 2014). In this model, ${T}_{0\mathrm{LV}}$ was chosen as the average of two literature measurements from Piroddi et al. (2007) and Racca et al. (2016), while ${t}_{0}$ was based on Mulieri et al. (1992) for an 800 ms cardiac cycle. FE model parameters are given in Table S1 in supplementary text.

### Optimality criteria

Biomechanics characteristics maps for stroke work, volume-averaged myofiber stress, deformational burden, and transmural strain variance as a function of helix angle configurations were generated. Equations on the computation of these values are given in the supplementary material. In this work, the optimal point on each characteristic map was considered to be the point with the maximum stroke work, the maximum volume-averaged myofiber stress, the minimum deformational strain energy density amplitude, and the minimum transmural strain variance.

### Calculation of FE versus image strain error

We compared FE global myocardial strains to that obtained from image tracking to investigate the range of helix angles that minimized the difference, so as to identify likely range of helix angles in our patient-specific cases. We defined FE versus image strain error (ER) as:

7 $$\mathrm{ER}={\left({\varepsilon }_{\mathrm{long},\mathrm{FE}}-{\varepsilon }_{\mathrm{long},\mathrm{echo}}\right)}^{2}+{\left({\varepsilon }_{\mathrm{circ},\mathrm{FE}}-{\varepsilon }_{\mathrm{circ},\mathrm{echo}}\right)}^{2},$$

where ${\varvec{\varepsilon}}$ is the end-systolic Green–Lagrange strain with end-diastole as the reference, and subscripts “long” and “circ” refers to longitudinal and circumferential strain directions, and subscripts “FE” and “echo” refer to the source of the strain values.

## Results

### FE and lumped parameter modeling for fetal subject Case 1

**Fig. 4:**
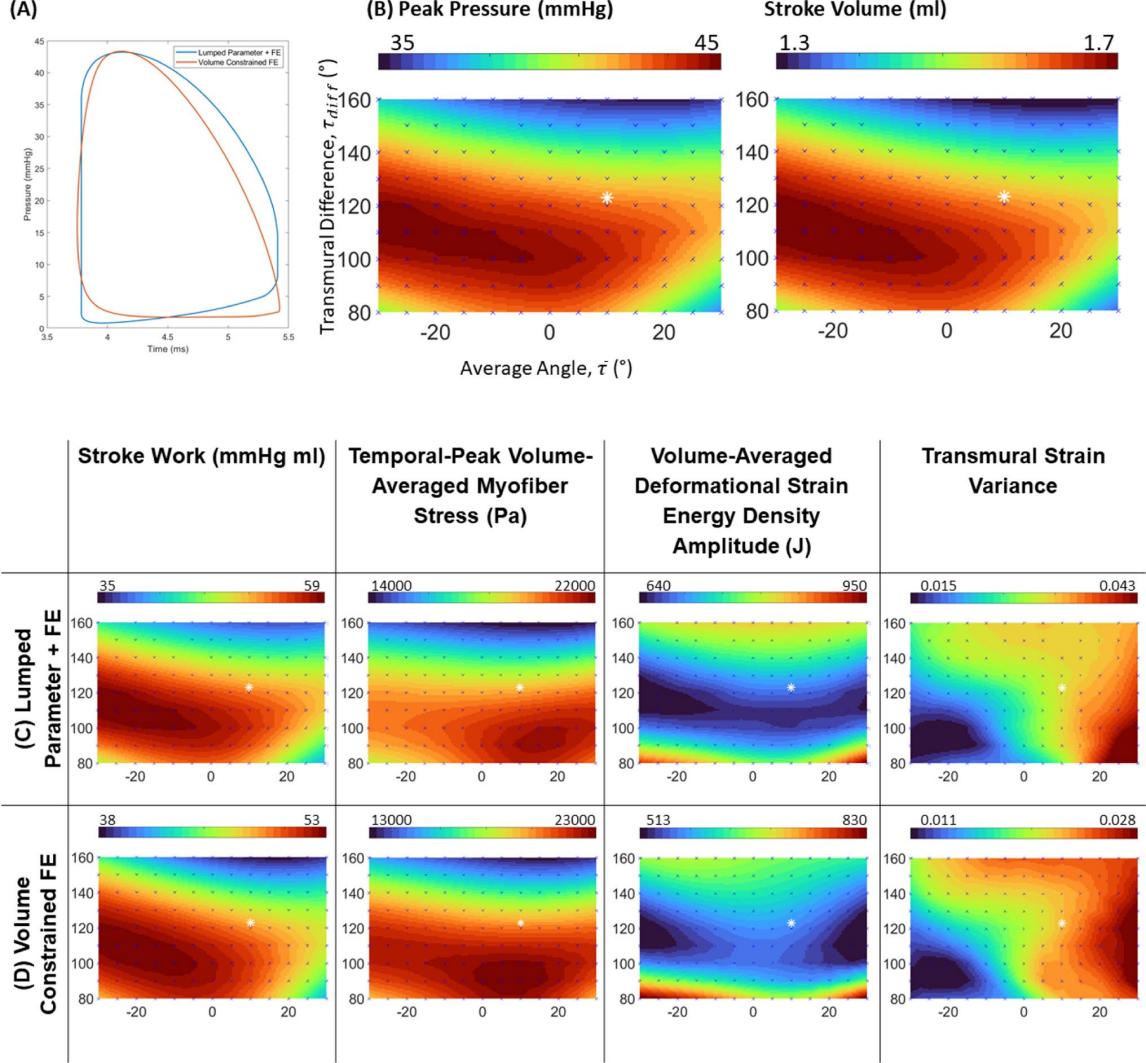
**A** Pressure–volume (PV) loops of fetal Case 1 LV obtained via the FE with lumped parameter and the volume-constrained FE approaches. **B**, **C** Maps of biomechanical characteristics obtained via the FE with lumped parameter approach, plotted as a function of helix angle configurations, for (**B**) peak LV pressure and stroke volume, and **C** stroke work, temporal-peak spatially averaged myofiber stress, deformational strain energy density amplitude, and transmural variability of fiber strains. **D** The same biomechanics characteristic maps as (**C**), but obtained via the volume-constrained FE approach, demonstrating similarity to that obtained from the FE with lumped parameter approach. Values on all maps were interpolated from points where data was obtained from simulations (indicated by blue crosses on maps) and all contour maps in this figure contain the same axes as (**B**). The white asterisk plots the average literature helix angle configuration of ($\overline{{{\uptau}}}\cong 1{0 }^{\mathbf{o}},{{{\uptau}}}_{{d}{i}{f}{f}}\cong {123}^{\mathbf{o}}$)

For fetal Case 1, at the helix angle configuration was $\overline{\tau }={0}^{\circ}$ and ${\tau }_{\mathrm{diff}}={120}^{\circ}$, the model was successfully tuned to achieve the expected physiology, a peak pressure of 43.24 mmHg and an end-diastolic pressure of 5 mmHg (Johnson et al. 2000), and a stroke volume of 1.64 ml, which was close to the measurements from 3D segmentation and motion tracking from the echo images (1.66 ml), as shown in Fig. 4A.

Simulations were further conducted over a range of helix angle configurations, and the results are shown in Fig. 4B, C, in the form of maps of biomechanical or cardiac function characteristics as a function of the helix angle configuration. Results show that helix angle configuration had substantial effects on these functional and biomechanical characteristics, given that the maps had substantial variations across $\overline{\tau }$ and ${\tau }_{\mathrm{diff}}$.

The maps for stroke work (work done by the heart or area within the PV loop), peak LV pressure, and LV stroke volume were very similar, suggesting that these outcomes were closely related. As such, we only need to consider one of these maps and could infer information about the remaining. Stroke work was high when ${\tau }_{\mathrm{diff}}$ was lower than 120°, but it quickly decreased above this cutoff. It had a decreasing trend with $\overline{\tau }$, and the spline interpolated peak and optimal point was found at ($\overline{\tau }=-29.93^\circ$, ${\tau }_{\mathrm{diff}}=113.24$).

Results further showed that the stress in the helix angle direction (“myofiber stress,” or stress in the “myofiber” direction) was also high when ${\tau }_{\mathrm{diff}}$ was lower than 120°, and it also quickly decreased above this cutoff, suggesting that the myocytes were best aligned with myocardial stresses when ${\tau }_{\mathrm{diff}}$ was lower than 120°. The spline interpolated peak and optimal point for myofiber stress was at ($\overline{\tau }=14.96^\circ$, ${\tau }_{\mathrm{diff}}=91.80^\circ$), which was at a positive $\overline{\tau }$, rather than a negative $\overline{\tau }$ where the stroke work was optimal.

To quantify the deformational burden suffered by the myocardium, we represented it with the amplitude of change in the volume-averaged deformational strain energy density from end-diastole to end-systole. This parameter signified the internal work of the LV or the amount of energy expended to deform the myocardium and could thus indicate the deformational burden. Here, a band of low values were found across a wide range of $\overline{\tau }$ at ${\tau }_{\mathrm{diff}}$ between 100° and 130^°^, suggesting that within this range of helix angle configurations, a lower amount of mechanical energy is needed to deform the myocardium over the cardiac cycle.

The transmural variability of myocardial strains was quantified by the variance of strain data (in the direction of helix angle, from end-systolic to end-diastolic) across a transverse plane at mid-ventricle. We find that generally, transmural strain variability increases with increasing $\overline{{\tau}}$. A minimum point appeared at the helix angle configuration of $(\overline{\tau }=-{21.01}^{\circ} ,{\tau }_{\mathrm{diff}}={94.43}^{\circ} )$.

Further, Fig. 4 compares the results from the volume-constrained FE simulations (Fig. 4D) to the FE with lumped parameter simulations (Fig. 4C), demonstrating that although there were some magnitude differences between the two approaches, the maps of how biomechanical characteristics varied with helix angle configurations were similar between the two approaches. The structural similarity index (SSIM) (Wang et al. 2004) of each type of map was calculated between the two approaches, using the FE with lumped parameter as the reference, and was found to be 0.983, 0.984, 0.908, and 0.900 for stroke work, peak myofiber stress, deformational burden, and transmural strain variance, respectively. All SSIM outputs were close to 1, suggesting high similarity (Wang et al. 2004). The PV loop of the volume-constrained approach also compared reasonably with that from the FE with lumped parameter approach (Fig. 4A), in terms of peak and minimum pressures and volumes and area within the PV loop. However, the volume-constrained FE models’ volume over time waveforms were extracted as a temporally smoothed waveform, where the isovolumetric regions were not produced, especially for isovolumetric contraction. Nonetheless, since our biomechanical maps (Fig. 4C, D) were not extracted from the isovolumetric regions, this did not affect our results. Further, since the volume-constrained approach is about 300 times less computationally expensive, we could use it for more extensive subsequent investigations.

### Patient-specific volume-constrained FE results

**Fig. 5:**
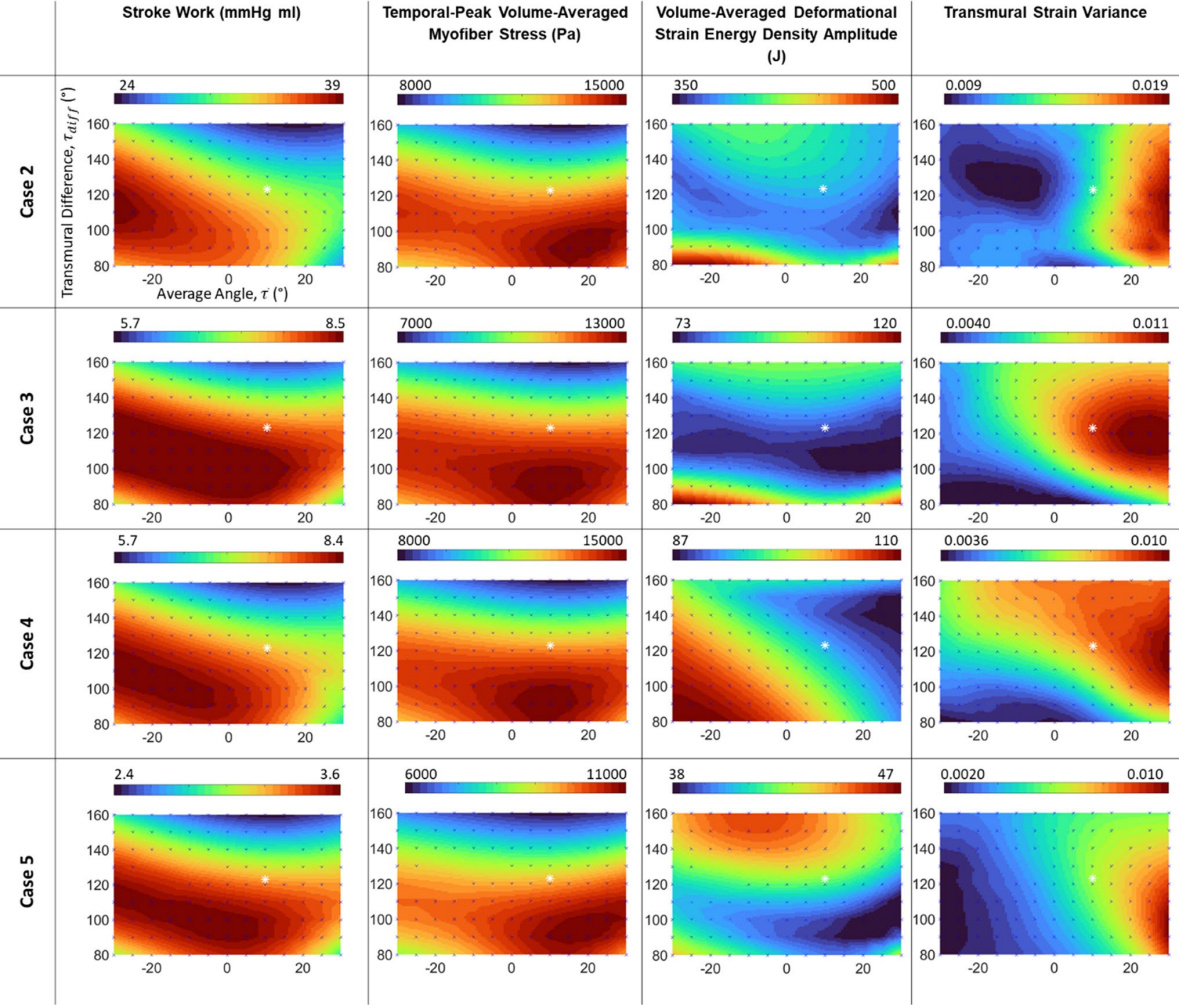
Maps of biomechanics characteristics plotted as a function of helix angle configurations for the patient-specific fetal LV cases, obtained via the volume-constrained FE simulation approach. Values on all maps were interpolated from points where data was obtained from simulations (indicated by blue crosses on maps), and all contour maps in this figure contain the same axes as top left. The white asterisk plots the average literature helix angle configuration ($\overline{{{\uptau}}}\cong 1{0 }^{\mathbf{o}},{{{\uptau}}}_{{d}{i}{f}{f}}\cong {123}^{\mathbf{o}}$)

Using the volume-constrained FE approach, we investigated the effects of helix angle configurations on the same set of LV biomechanics characteristics for 4 other fetal subjects, as shown in Fig. 5. We found that the results above were largely similar to the results presented in Fig. 4. The only exception was the deformational burden map for Case 4, which demonstrated a decreasing trend with increasing $\overline{\tau }$ and ${\tau }_{\mathrm{diff}}$. This difference was likely due to geometric differences between this LV from the others, where the Case 4 LV was more symmetric, compared to other cases in which the apex leaned medially toward the septum. In our subsequent investigations with idealized LV geometries, similar differences were observed between the “Symmetric” and the asymmetric “Regular” LV models. (Note: the range of helix angles investigated in the idealized geometries was wider.)

In these patient-specific biomechanics characteristics map results, the optimal point for myofiber stress was within the range of helix angles investigated for all cases and averaged to be at $(\overline{\tau }={13.57}^{\circ} \mathrm{and} {\tau }_{\mathrm{diff}}={91.15}^{\circ} )$ across the 5 patient-specific cases. However, for other characteristics, the optimal point was not within the range of angles for some cases.

To determine if the fetal heart helix angle is close to any of the optimality points on these maps, we used literature reported fetal helix angle configurations, which ranged from $({0}^{\circ} <\overline{\uptau }<{25}^{\circ} ,{110}^{\circ} <{\uptau }_{\mathrm{diff}}<{150}^{\circ} )$, (Ohayon et al. 1999; Garcia-Canadilla et al. 2018; Nishitani et al. 2020) and averaged as $(\overline{\uptau }\cong 1{0 }^{\circ} ,{\uptau }_{\mathrm{diff}}\cong {123}^{\circ} )$, and marked this point on the biomechanical maps (Figs. 4 and 5). From these figures, it could be observed that this average helix angle configuration corresponded to high myofiber stress and moderately high stroke work but was not at the optimality point for either parameter. Further, it was close to or within the region of low deformational strain energy density amplitude. However, in terms of transmural strain variability, it achieved a moderate to moderately high value, across the contour variation. We thus concluded that the reported helix angle configuration did not achieve optimality in any of the biomechanical parameters we investigated but achieved parameter values close to the optimal value for some parameters. Since the helix angle configuration was closest to the peak myofiber stress optimality point, we hypothesize that myofiber stress was the most important stimulus for fetal heart helix angle remodeling.

### Verification of helix angle configurations with echo-measured strains

**Fig. 6:**
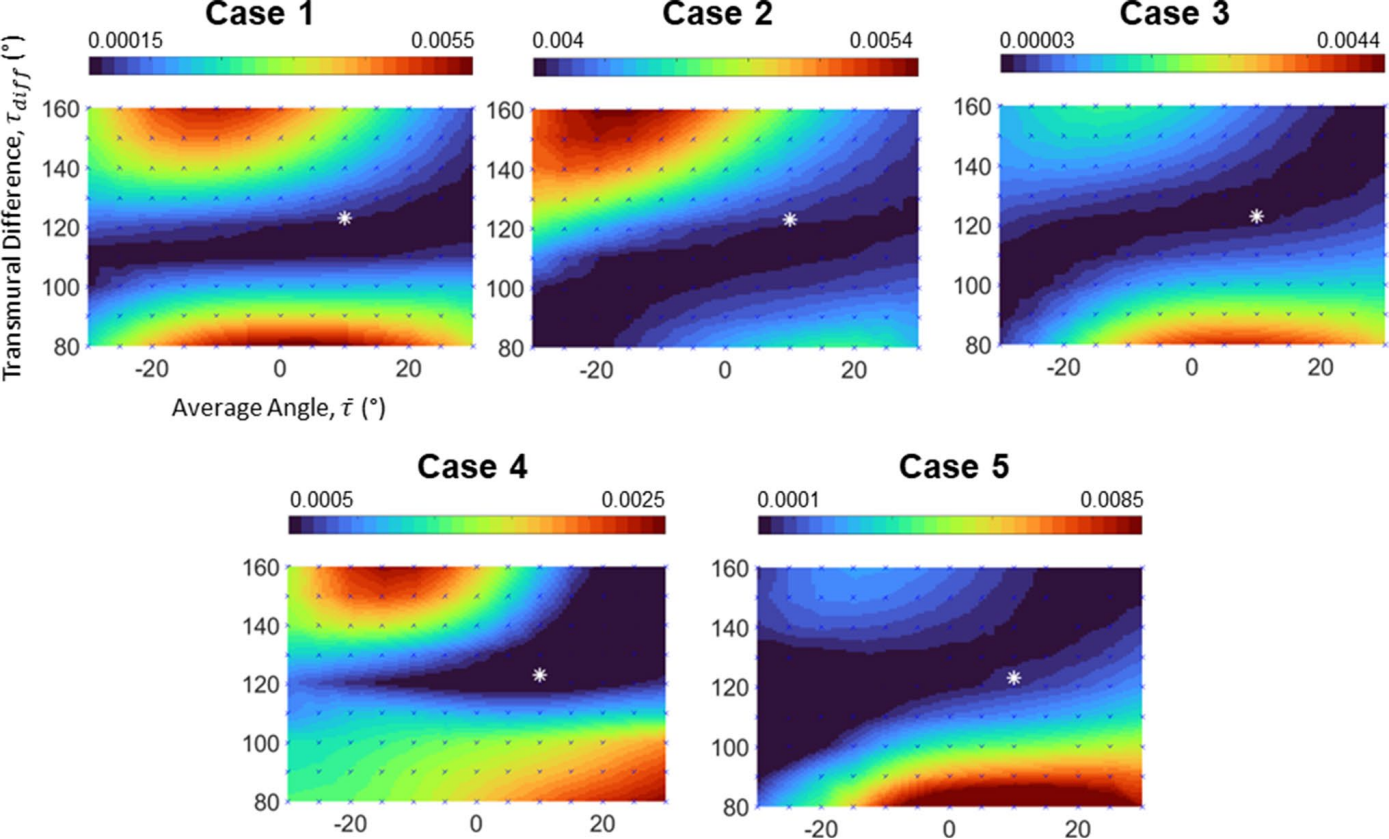
Strain comparison errors in the comparison of strains derived from FE and that derived from echo images, according to Eq. 7. Values on all maps were interpolated from points where data were obtained from simulations (indicated by blue crosses on maps), and all contour maps in this figure contain the same axes as Case 1. The white asterisk plots the average literature helix angle configuration of ($\overline{{{\tau}}}\cong 1{0 }^{{\varvec{o}}},{{{\tau}}}_{{{d}}{{i}}{{f}}{{f}}}\cong {123}^{{\varvec{o}}}$)

Next, we examined which helix angle configurations had the smallest errors between FE simulated myocardial strains and image-measured strains. Results are shown in Fig. 6, demonstrating how the zone of ${\tau }_{\mathrm{diff}}$ between 110° and 130° had consistently low strain comparison errors and was likely to be the actual helix angle configurations as strain errors elevated beyond this range. However, a range of $\overline{{\tau}}$ could not give a similarly satisfactory match between FE and image strains and could not assist in narrowing the range of plausible helix angle configurations. This was due to the strain characteristics being more sensitive to ${\tau }_{\mathrm{diff}}$ and less sensitive to $\overline{\tau }$. However, visual evaluation of the myocardial motions at different $\overline{\tau }$ revealed that varying $\overline{\tau }$ altered the extent of LV torsional motion mildly, even if it did not significantly change peak longitudinal and circumferential strains. When we investigated the maps for longitudinal or circumferential strain errors individually instead of together, we found that longitudinal strain errors were generally low at mid-range ${\tau }_{\mathrm{diff}}$, while circumferential strain errors were generally low at mid to high ${\tau }_{\mathrm{diff}}$, but both were similarly insensitive to $\overline{\tau }$, as demonstrated in supplementary Fig. S4.

Given that experimental evaluations in the literature typically concluded that $\overline{\tau }$ was between 0° and 25° (Ohayon et al. 1999; Garcia-Canadilla et al. 2018; Nishitani et al. 2020), it was likely that the actual helix angle configurations were $\left({0}^{\circ} <\overline{\tau }<{25}^{\circ} , {110}^{\circ} <{\tau }_{\mathrm{diff}}<{130}^{\circ} \right)$. Table 1 lists the helix angle configurations that provided the closest strains to image-derived strains and showed that almost all the strains were sufficiently close, within a margin of errors.

**Table 1:** The helix angle configuration that provided FE-derived strains closest to the image-derived strains and comparison between strains derived from both modalities

	Best matching helix angle configuration		Circumferential strain		Longitudinal strain	
Case	$\overline{\tau }$	${\tau }_{\mathrm{diff}}$	Image (%)	Simulation (%)	Image (%)	Simulation (%)
1	− 20°	110°	8.25	9.05	8.95	8.98
2	− 20°	100°	10.90	10.83	7.20	7.30
3	− 25°	110°	13.60	13.88	11.55	12.03
4	25°	140°	14.76	13.31	12.20	11.81
5	− 30°	110°	10.10	12.92	11.25	12.22

### Effects of LV geometry on the relationship between helix angle and biomechanics characteristics

**Fig. 7:**
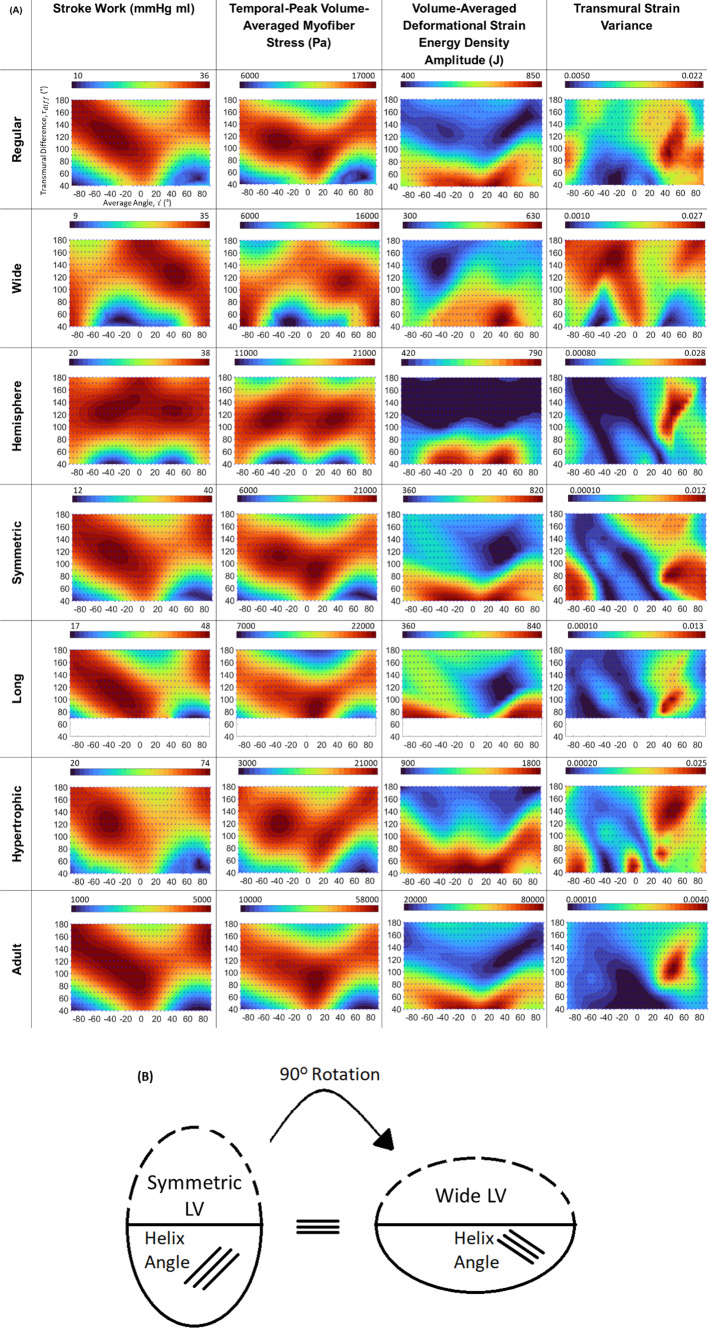
**A** Maps of biomechanics characteristics plotted as a function of helix angle configurations, for the various idealized LV geometries (shown in Fig. 3), where values on all maps were interpolated from points where data was obtained from simulations (indicated by blue crosses on maps) and all contour maps in this figure contain the same axes as top left. **B** Schematic of the prolate shapes that represented the “Symmetric” and “Wide” LV geometries, demonstrating that these are similar configurations but rotated by 90°

To understand why helix angle configurations had specific influence on biomechanics characteristics as shown in Figs. 4 and 5, we repeated the volume-constrained FE simulations on conceptualized and idealized LV geometries of extreme dimensions, shown in Fig. 3. We first tested whether LV shapes, including aspect ratios, wall thickness, and LV shape symmetry, affected the influence of helix angle configuration on biomechanics. Results presented in Fig. 7A showed that the maps of biomechanics characteristics over various helix angle configurations were changed drastically when the LV geometry was altered. For the “Long” geometry, results for ${\tau }_{\mathrm{diff}}$ below 70° are not shown as there was difficulty in FE simulation convergence for this extremely elongated geometry.

The comparison of the “Regular” and “Symmetric” geometries could test the effects of LV shape symmetry. These geometries produced similar stroke work and myofiber stress variation maps. However, the deformational burden was greatly affected by the geometry change. The “Regular” geometry produced the horizontal band of low values similar to that noted in patient-specific cases with asymmetric geometry (all cases except for Case 4), whereas the “Symmetric” geometry resulted in a low region at ${(20}^{\circ} <\overline{\uptau }<{60}^{\circ} , {100}^{\circ} <{\uptau }_{\mathrm{diff}}<{160}^{\circ} )$, similar to that observed in patient-specific case 4, which had a more symmetric geometry.

The “Wide,” “Hemisphere,” “Symmetric,” and “Long” geometries represented a gradual increase in aspect ratio (ratio of longitudinal to lateral dimensions). From the results, gradual shifting of biomechanics characteristics patterns with the increase in aspect ratio could be observed. In particular, the “Wide” and “Regular” geometries had aspect ratios that were reciprocals of each other, with their longitudinal and lateral dimensions switched over. As shown in Fig. 7B, the “Wide” case represented a prolate that was rotated 90° from the prolate represented by the “Symmetric” case. This meant that these two cases should have similar biomechanics function map if all the helix angles of one case were offset from the other case by 90°. From the stroke work, deformational burden and myofiber stress results, the biomechanics function maps of the two geometries indeed appeared to have similar patterns that were offset by 90° in $\overline{\tau }$ from each other (offset in the horizontal axis direction). These results demonstrated that LV geometry was an important determining factor of these biomechanics characteristic patterns.

Comparing the “Symmetric” and the “Hypertrophic” cases, we found visible but minor differences, and that the optimal points in these biomechanical characteristics had shifted, suggesting that alterations in wall thickness can also affect the influence of helix angles on functionality and shift the optimality of the heart’s function.

In terms of transmural strain variability, a range of configurations led to low variability; however, in the “Regular” case, the helix configuration of $\left(\overline{\tau }\cong {-29}^{^\circ }, {\tau }_{\mathrm{diff}}\cong {51}^{^\circ }\right)$ appeared to be the optimal point. However, with geometric shapes, the maps varied, and the optimal point shifted drastically, again demonstrating the strong influence of LV geometry.

To understand if the size of the LV would affect the biomechanics function maps, we compared FE results of the adult “Regular” geometry to fetal “Symmetric” geometry, as they have the same symmetric geometry. We found only minor differences in surface variation for stroke work, myofiber stress, and deformational burden. This suggested that size scaling the LV from fetal to adult sizes did not play a role in determining the effects of helix angle configurations on functionality, or in the biomechanical optimality of the heart.

Interestingly, when we perform simulations of the “Symmetric” case with an endocardial-to-epicardial inversion of the helix angles, such that endocardial helix angle is positive and epicardial helix angle is negative instead of the other way round, the biomechanical maps were not significantly altered, as shown in supplementary Fig. S5. The directionality of helix angle changes across the LV wall thickness was thus not consequential to the biomechanical characteristics. Rather, it was the overall helix angle configuration of the whole thickness of the LV wall that mattered.

## Discussion

In the current study, we conducted image-based FE simulations on both realistic patient-specific LV geometries and idealized LV geometries to understand the contribution of helix angle configurations on the biomechanical function of the fetal heart and to propose a plausible range of helix angle configurations to inform future simulation studies. We also investigated which helix angles would give strains matching those observed in echo images and studied the effects of LV size and shapes on the relationship between helix angles and biomechanics.

Past studies had found that helix angle had substantial impact on cardiac biomechanics in adult hearts and found that known helix angles from the literature were close to functional optimality. Vendelin et al. (2002) and Rijcken et al. (1999) had conducted such studies and proposed that the homogeneity of strains and stresses was two such optimality conditions. Further, Pluijmert et al. (2012) and Washio et al. (2020) simulated adaptive helix angle remodeling according to biomechanical stimuli, reorientating the local helix angle to the local principal direction of strain in the former, and to the local principal direction of stress in the latter. They found that these biomechanics-induced remodeling produced realistic helix angle configurations, suggesting that biomechanics played a role in helix angle remodeling. Investigations in adult sheep animals, however, conflicted with results by Vendelin et al. (2002) and found that histologically measured helix angles did not coincide with the optimality configurations (Ennis et al. 2008).

In our study, we found that helix angle was similarly influential on the biomechanics of the fetal heart. Our study, however, departed from past optimality studies’ approach of using idealized LV geometry (Rijcken et al. 1999; Vendelin et al. 2002), or using only a single patient-specific case (Palit et al. 2015), and we extended testing to several image-based patient-specific fetal LV geometries. Further, we investigated a different set of biomechanical parameters for optimality. While Vendelin et al. (2002) and Rijcken et al. (1999) advocated for the homogeneity of strains and stresses as the biomechanics optimality, we had found that this criterion was not optimized in our patient-specific fetal LV geometries at the literature reported helix angle configurations, or at the range of helix angle configurations found to have FE strains matching those from our echo images. This was likely due to differences in LV geometries in our study compared to theirs, as we used patient-specific fetal geometries, while they used idealized adult geometries.

Instead, we find that the criteria that were somewhat optimized were low deformational burden, high stress in the helix angle direction, and to a lesser extent, high work done by the LV. We propose that it is reasonable to expect the LV to remodel its helix angle configuration toward one that would maximize pumping work output, given that this is the primary function of the heart, although the feedback mechanism that leads to this remodeling is currently unclear. We also propose that high deformational burden on myocardium tissue can lead to excessive microstructural damage that imposes high metabolic burden for which the cells undergo tissue repair, and thus, it would be reasonable to expect low deformational burden to be an optimality criterion for cardiac development. We further propose high myofiber stress to be a reasonable biomechanical optimality criterion, as it indicates a full engagement of the myocytes to produce contractile forces in the direction of the highest stresses. This optimality criterion is corroborated by the work of Washio et al. (2020), who found that anticipating myocyte helix angle to remodel toward the direction of highest stress would yield a physiological remodeling outcome. We believe that future work testing these proposals is warranted.

However, we noted that none of the commonly reported fetal heart helix angles coincided with the optimal points of these three biomechanical parameters. Since the optimality points for these various parameters investigated did not coincide with each other, it was not possible to concurrently achieve optimality in all these parameters. However, since the literature reported helix angles of $(\overline{\tau }\cong {10}^{\circ} ,{\tau }_{\mathrm{diff}}\cong {123}^{\circ} )$ appeared to be in the middle of these optimal points, we hypothesize that the fetal heart remodels toward all of these biomechanical criteria concurrently and achieves semi-optimality in all of them, but true optimality in none. Again, future work testing this hypothesis is warranted.

Comparing our FE myocardial longitudinal and circumferential strains to those measured from the echocardiography images, we found that a helix angle configuration of ${(0}^{\circ} <\overline{\tau }<{25}^{\circ} ,{110}^{\circ} <{\tau }_{\mathrm{diff}}<{130}^{\circ} )$ could enable a good match between FE strains and image strains and therefore we recommend this configuration for future fetal heart simulation studies. The approach we used here could also be a basis for future work that enables the back-computation of helix angles from clinical images.

Another interesting outcome of our study was the discovery that the biomechanical optimality maps were essentially a response to LV geometry and were sensitive to changes in the LV geometry, including the symmetry of the geometry, wall thickness, and aspect ratio. This would suggest that the biomechanical optimality of the LV can change with shape and helix angle remodeling, such as during fetal cardiac dysfunction (Torre et al. 2014) and congenital heart malformations (Tulzer et al. 2021). Since we have found that the optimality did not change with cardiac size, it seems reasonable to expect that morphological changes due to disease would also shift cardiac biomechanics optimality in adult hearts, such as during heart failure (Nauta et al. 2020). We believe that these notions warrant further investigations.

There are several limitations to our study. Firstly, we adopted the idealization that the helix angle varied linearly from the epicardium to the endocardium, and that the same transmural helix angle configuration applies to any location on the LV wall. Although most experimental studies found that the linear transmural variation be true through the entire LV wall (Nishitani et al. 2020) or bulk of the myocardium (Rohmer et al. 2007), some studies reported nonlinear transmural variation (Garcia-Canadilla et al. 2018), and several studies have reported that different regions of the heart have varied helix angle configurations (Ennis et al. 2008; Garcia-Canadilla et al. 2018). Further, we did not investigate myocardium sheetlet sliding effects (Nielles-Vallespin et al. 2017). These idealizations could have led to errors in our results. Secondly, the right ventricle, which could have affected the LV biomechanics, was not included in our FE simulations. Finally, our use of the volume-constrained FE approach relied on smoothed volume over time waveforms and approximated but did not show isovolumetric behavior in the PV loop.

## Conclusion

We performed FE simulations of the fetal LV and found that helix angle configurations had substantial influence on fetal heart biomechanics and function. Experimentally measured fetal heart helix angle configurations from the literature achieved close to optimal values in maximizing LV work done, maximizing stress in the helix angle orientation, and minimizing the deformational burden of the myocardial tissue, but were not at the optimal configuration of any of these criteria. The closest match in all cases was toward maximizing stress in the helix angle orientation, thus suggesting this to be a main stimulus for helix angle remodeling, but not solely responsible. The literature reported helix angle configuration enabled a close match in the myocardial strains measured from our echo images and that obtained from our FE modeling, providing some validation for the configuration. Further, the relationship between helix angle configurations and biomechanical outcomes was brought about by the “Asymmetric” geometry of the fetal LV and was sensitive to geometric changes, including wall thickness and LV chamber aspect ratio, but was not sensitive to size scale of the LV, when comparing the fetal “Symmetric” and adult “Regular” geometries. This suggested that the discussion of changes in biomechanical optimality due to disease is applicable to the adult LV also.

## Supplementary Information

Below is the link to the electronic supplementary material.

Supplementary file1 (DOCX 1729 KB)

Supplementary file2 (MP4 257 KB)
