# Supplementary material for: The dependency of fetal left ventricular biomechanics function on myocardium helix angle configuration
Source: Biomech Model Mechanobiol. 2022 Dec 22;22(2):629–43. doi: 10.1007/s10237-022-01669-z (PMC10097781; doi:10.1007/s10237-022-01669-z)
Supplement: Supplementary file 1 — Supplementary file1 (DOCX 1729 KB) [file 10237_2022_1669_MOESM1_ESM.docx]

**Supplementary Material**

**Table S1.** FE Modelling parameters for all cases.

| Parameter | Symbol | Unit | Fetal Idealised Models | Adult Idealised Model | 32-Week | | 22-Week | | |
| --- | --- | --- | --- | --- | --- | --- | --- | --- | --- |
|  |  |  |  |  | Case 1 | Case 2 | Case 3 | Case 4 | Case 5 |
| Time to peak tension | $t_{0}$ | ms | 140.5 | 172 | 140.5 | 140.5 | 140.5 | 140.5 | 140.5 |
| Maximum tension | $T_{0LV}$ | Pa | 40,000 | 91,600 | 52,000 | 40,000 | 26,000 | 30,000 | 33,000 |
| Sarcomere length under no active tension | $l_{0}$ | $\mu m$ | 1.58 | 1.58 | 1.58 | 1.58 | 1.58 | 1.58 | 1.58 |
| Relaxed sarcomere length | $l_{r}$ | $\mu m$ | 1.85 | 1.85 | 1.85 | 1.85 | 1.85 | 1.85 | 1.85 |
| Time intercept of linear relaxation duration with sarcomere length | *b* | ms | -835 | -1600 | -830 | -800 | -805 | -835 | -835 |
| Gradient of linear relaxation duration with sarcomere length relation | *m* | $ms\mu m^{-1}$ | 524 | 1049 | 524 | 524 | 524 | 524 | 524 |
| Maximum intracellular calcium concentration | ${Ca}_{0}$ | $\mu M$ | 4.35 | 4.35 | 4.35 | 4.35 | 4.35 | 4.35 | 4.35 |
| Passive stiffness coefficient | C | Pa | 100 | 100 | 100 | 100 | 100 | 100 | 100 |
| Stiffness coefficient in fiber direction | $b_{ff}$ | - | 29.9 | 29.9 | 29.9 | 29.9 | 29.9 | 29.9 | 29.9 |
| Stiffness coefficient in sheet and sheet normal direction | $b_{xx}$ | - | 13.3 | 13.3 | 13.3 | 13.3 | 13.3 | 13.3 | 13.3 |
| Stiffness coefficient in shear directions | $b_{fx}$ | - | 26.6 | 26.6 | 26.6 | 26.6 | 26.6 | 26.6 | 26.6 |
| Cardiac cycle | *BCL* | ms | 400 | 800 | 400 | 400 | 400 | 400 | 400 |

***Table S2.*** Modelling parameters for the fetal lumped-parameter model (Pennati et al. 1997).

|  | **Parameter** | **Description** | **Value** |
| --- | --- | --- | --- |
| **Capacitors** | AAC | Ascending aorta | 0.05 |
|  | A01C | Aortic arch | 0.08 |
|  | A02C | Thoracic descending aorta | 0.07 |
|  | A03C | Abdominal descending aorta | 0.04 |
|  | A04C | Femoral descending aorta | 0.05 |
|  | PA1C | Main pulmonary artery | 0.08 |
|  | PA2C | Pulmonary artery | 0.08 |
|  | LUNGC | Lung | 0.40 |
|  | CAC | Cerebral arteries | 0.07 |
|  | BRC | Brain | 0.3 |
|  | UBC | Umbilical | 0.85 |
|  | INTEC | Intestinal circulation | 0.25 |
|  | KIDC | Kidney | 0.02 |
|  | HEC | Liver | 3.00 |
|  | PLACC | Placenta | 1.50 |
|  | LEGC | Leg | 4.00 |
|  | UVC | Umbilical vein | 0.30 |
|  | SVCC | Superior vena cava | 1.00 |
|  | IVCC | Inferior vena cava | 0.60 |
|  | RAC | Right atrium | 1.00 |
|  | LAC | Left atrium | 2.00 |
| **Inductors** | DAL | Ductus arteriosus | 0.006 |
|  | AO1CAL | Aortic arch to cerebral arteries | 0.08 |
|  | AAAO1L | Ascending aorta to aortic arch | 0.002 |
|  | PA1PA2L | Main pulmonary artery to pulmonary artery | 0.02 |
|  | RARAVALVL | Right atrium to tricuspid valve | 0.0016 |
|  | LALAVALVL | Left atrium to mitral valve | 0.0 |
| **Resistors** | AAAO1R | Ascending aorta to aortic arch | 0.12 |
|  | AO1AO2R | Aortic arch to thoracic descending aorta | 0.4 |
|  | AO2AO3R | Thoracic descending aorta to abdominal descending aorta | 0.04 |
|  | AO3AO4R | Abdominal descending aorta to femoral descending aorta | 0.06 |
|  | PA1PA2R | Main pulmonary artery to pulmonary artery | 0.07 |
|  | PA2LUNGR | Pulmonary artery to lung | 13.50 |
|  | DAR |  | 0.01 |
|  | AO1CAR | Aortic arch to cerebral arteries | 0.30 |
|  | CABRR | Cerebal arteries to brain | 3.00 |
|  | BRSVCR | Brain to superior vena cava | 8.50 |
|  | AO1UBR | Aortic arch to umbilical cord | 8.00 |
|  | UBSVCR | Umbilical cord to superior vena cava | 4.90 |
|  | AO3HER | Abdominal descending aorta to liver | 81.00 |
|  | AO3INTER | Abdominal descending aorta to intestinal circulation | 34.00 |
|  | INTEHER | Intestinal circulation to liver | 7.00 |
|  | AO3KIDR | Abdominal descending aorta to kidney | 3.50 |
|  | KIDIVCR | Kidney to inferior vena cava | 14.00 |
|  | AO4PLACR | Femoral descending aorta to placenta | 3.90 |
|  | PLACUVR | Placenta to umbilical vein | 3.40 |
|  | AO4LEGR | Femoral descending aorta to leg | 3.50 |
|  | LEGIVCR | Leg to inferior vena cava | 0.60 |
|  | UVHER | Umbilical vein to liver | 0.50 |
|  | HEIVCR | Liver to inferior vena cava | 0.16 |
|  | DVR | Ductus venosus | 1.30 |
|  | SVCRAR | Superior vena cava to right atrium | 0.20 |
|  | IVCRAR | Inferior vena cava to right atrium | 0.12 |
|  | LUNGLAR | Lung to left atrium | 2.00 |
|  | RARAVALVR | Right atrium to tricuspid valve | 0.00 |
|  | RVRVVALVR | Right ventricle to pulmonary valve | 0.08 |
|  | LVLVVALVR | Left ventricle to aortic valve | 0.00 |
|  | LALAVALVR | Left atrium to mitral valve | 0.00 |
| **K and B Relationship Terms** | FOK | Foramen ovale | 0.40 |
|  | DAK | Ductus arteriosus | 0.009 |
|  | DVK | Ductus venosus | 0.26 |
|  | RARAVALVK | Right atrium to tricuspid valve | 0.002 |
|  | RVRVVALVK | Right ventricle to pulmonary valve | 0.001 |
|  | LALAVALVK | Left atrium to mitral valve | 0.002 |
|  | LVLVVALVK | Left ventricle to aortic valve | 0.001 |
|  | FOB | Foramen ovale | 0.625 |
|  | DAB | Ductus arteriosus | 2.00 |
|  | DVB | Ductus venosus | 2.00 |
|  | RARAVALVB | Right atrium to tricuspid valve | 2.00 |
|  | RVRVVALVB | Right ventricle to pulmonary valve | 2.00 |
|  | LALAVALVB | Left atrium to mitral valve | 2.00 |
|  | LVLVVALVB | Left ventricle to aortic valve | 2.00 |

**Resistance and Compliance Scaling for Fetal Lumped Parameter Model**

Following Pennati et al.’s (1997) original description of the fetal circulatory system, we applied a further overall resistance scale factor of 1.37 to all resistances and an overall capacitance scale factor of 0.14 to all capacitances. Part of this scaling accounted for the age difference between Pennati et al’s model and our fetal case, and part of it was to cater for patient-specific characteristics such as ventricular size and stroke volume. This was done to enable our LV case to match the peak systolic pressure measured by Johnson et al. (2000) and the descending aorta pulse pressure measured by Versmold et al. (1981), both measured in humans. With our recalibrated model, the LV peak systolic pressure matched literature value to within 1.8% error (Johnson et al. 2000) and descending aorta pulse pressure matched literature values to within 2.1% error (Versmold et al. 1981). Figure S1 provides examples of pressure and flowrate tracings extracted from the lumped parameter model at $\bar{\tau}=0^{o},\tau_{diff}={120}^{o}$, to demonstrate physiological waveforms were achieved.


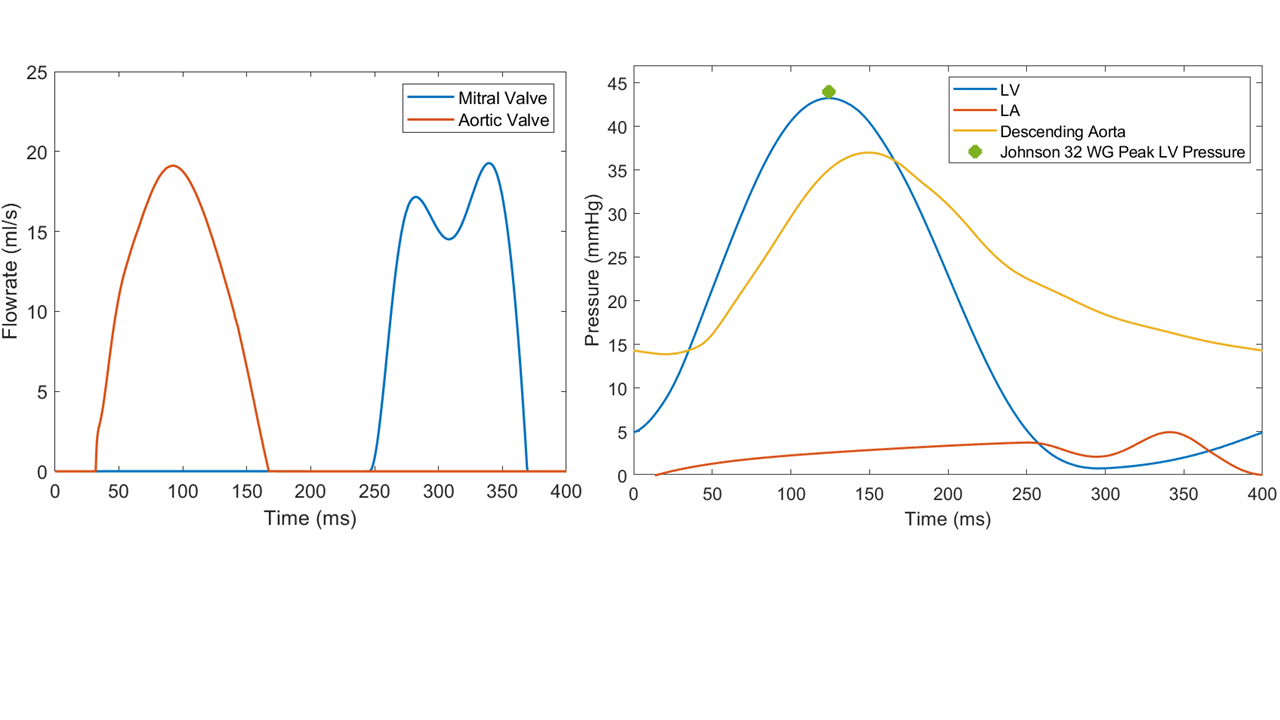


**Figure S1.** Case 1 flowrate and pressure tracings from the lumped parameter + FE model at $\bar{\tau}=0^{o},\tau_{diff}={120}^{o}.$ [Left] Mitral and aortic valve flowrates. [Right] LV, LA and descending aorta pressure variation throughout the cardiac cycle. LV showing a good peak pressure match to Johnson et al. (2000) measurements for a 32 WG LV.

**Computation of Biomechanics Characteristics**

Stroke Work referred to the work done by the LV on ejected fluid and was calculated as the area within the PV loop. Temporal-peak volume-averaged myofiber stress was calculated by analysing the FE results at peak systole time point with the highest pressure, taking the component of the stress tensor in the direction of the helix angle, and performing volume averaging, as shown in equation S1:

| $Myofiber Stress=\frac{1}{V}\int_{V} f_{d}\boldsymbol{\sigma}f_{d}dV$ | equation S1 |
| --- | --- |

Where V is is the volume of the myocardium, $f_{d}$ is unit vector in the myofiber direction, and $\boldsymbol{\sigma}$ is the stress tensor. Volume-averaged deformational strain energy density amplitude (deformational burden) was calculated using equation S2, where we first calculated the volume averaged strain energy density at every time point, and then found the difference between its highest value and its lowest value:

| $strain energy density amplitude=\max\left( \frac{\int_{V} W(t)dV}{V} \right)-\min\left( \frac{\int_{V} W(t)dV}{V} \right)$ | equation S2 |
| --- | --- |

Where W is the strain energy density function, and t is the time. Transmural strain variance was obtained by first calculating the minimum eigenvalue ($\lambda_{min}$) of the strain tensor, which signifies the largest principal contractile strain, and then calculating its standard deviation across a transverse cross-section at the mid-ventricular region, according to equation S3:

| $strain variance= \sqrt{\frac{\sum\left\vert\lambda_{min}-\bar{\lambda_{min}} \right\vert^{2}}{n}}$ | equation S3 |
| --- | --- |

**Transverse Angle Sensitivity Analysis**

To test if the specification of myofiber transverse angle will affect our results, we conducted a sensitivity analysis. We used the Vendelin et al. (2002) model, where transverse angles were 0° at the endocardium and epicardium and peak in the midwall, where the midwall angle linearly varied from positive to negative from the base to apex. We used two different magnitudes of transverse angles, as shown in figure S2A.

Results showed that both models of transverse angle did not produce biomechanical maps that were significantly different from simulations without the transverse angle (Figure S2B, C).


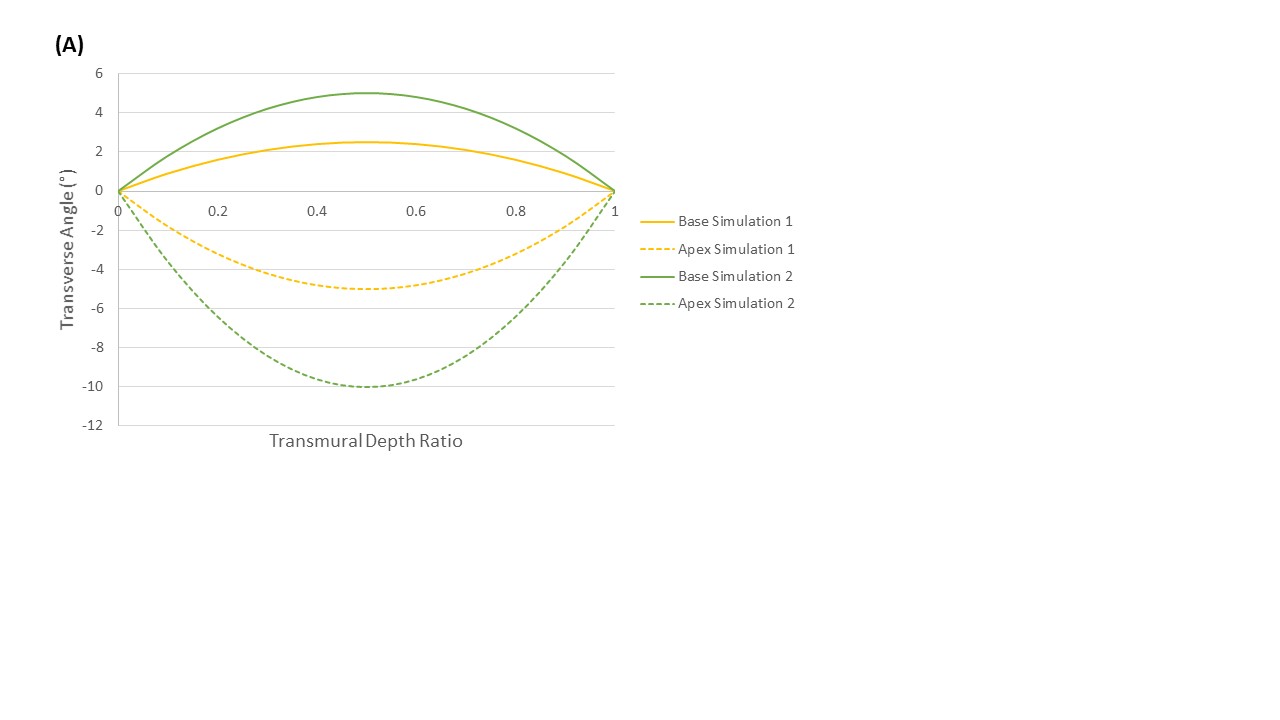


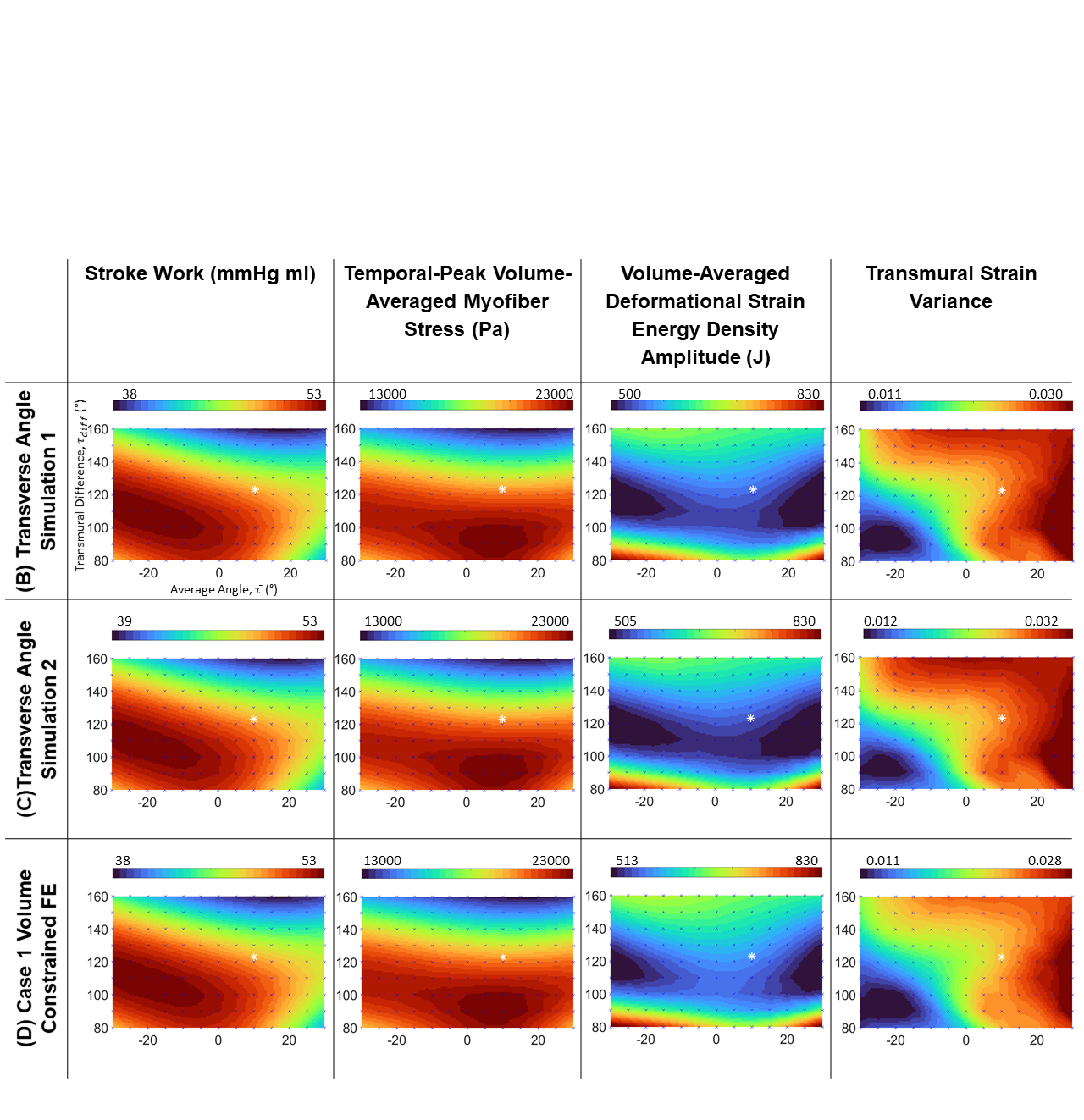


**Figure S2.** (A) Transverse angle versus transmural depth ratio (from 0 at endocardium to 1 at epicardium) for 2 scenarios, Simulation 1 and 2. Plots are shown for basal and apical regions, and a linear variation is assumed from base to apex. (B & C) Biomechanics maps of Fetal Case 1 with the 2 scenarios of transverse angles in (A) applied, compared to (D) without any transverse angle applied. Values on all maps were interpolated from points where data was obtained from simulations (indicated by blue crosses on maps) and all contour maps in this figure contain the same axes as top left. The white asterisk plots the average literature helix angle configuration ($\bar{\boldsymbol{\tau}}\boldsymbol{\cong1}\boldsymbol{0}^{\boldsymbol{o}}\boldsymbol{,}\boldsymbol{\tau}_{\boldsymbol{diff}}\boldsymbol{\cong}\boldsymbol{123}^{\boldsymbol{o}}$).

**PV Loops Examples for All Fetal Models**

Examples for the PV loops for all remaining fetal model simulations at $\bar{\tau}$ = 0° and $\tau_{diff}$ = 120°, using the volume-constrained FE methods, are shown in Figure S3.


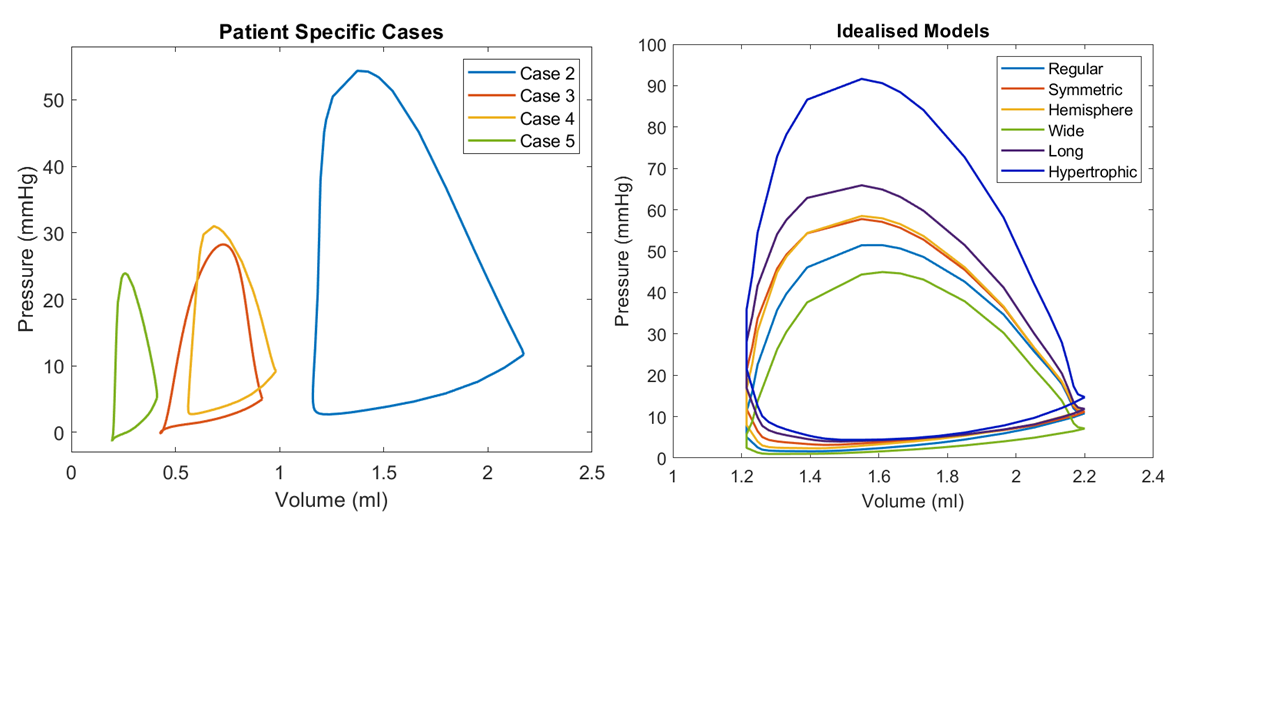


**Figure S3.** PV loops for LV models investigated for the helix angle configuration of $\bar{\tau}$ = 0° and $\tau_{diff}$ = 120°, using volume-constrained FE.


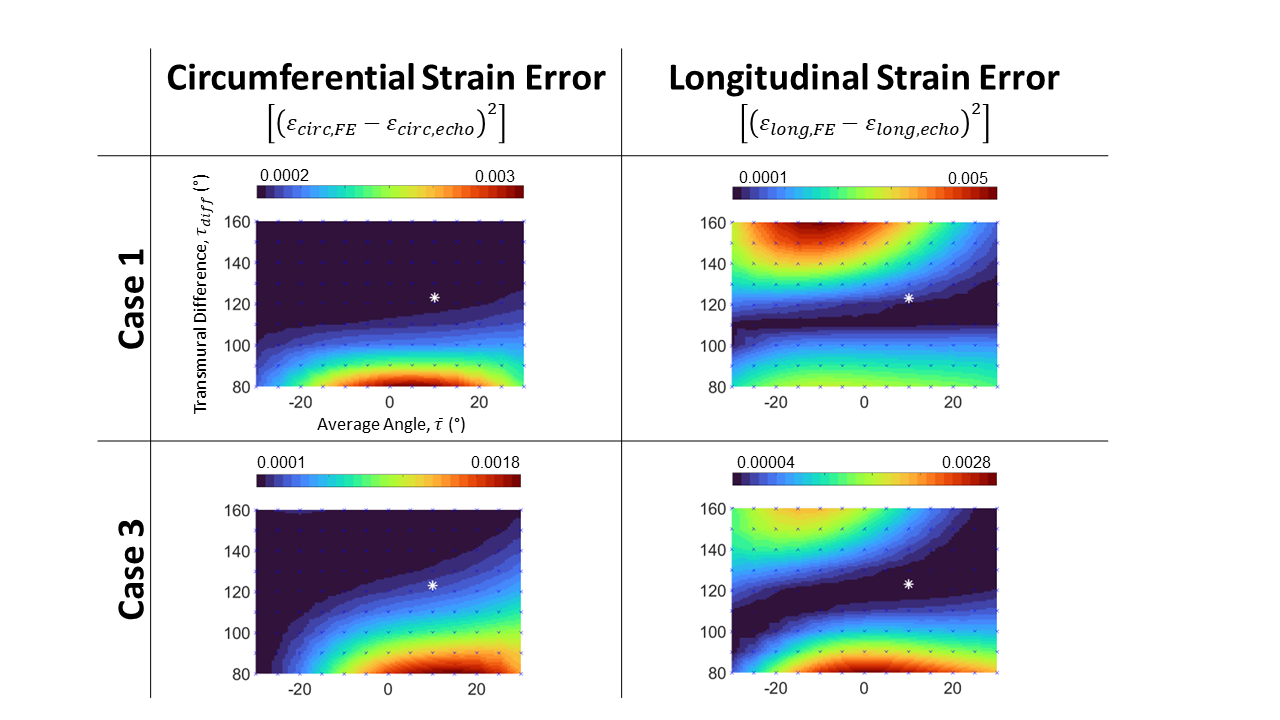


**Figure S4.** Error between Image (echo) and simulation (FE) derived circumferential ($\varepsilon_{circ}$) and longitudinal ($\varepsilon_{long}$) strains, for Case 1 and Case 3. Values on all maps were interpolated from points where data was obtained from simulations (indicated by blue crosses on maps) and all contour maps in this figure contain the same axes as top left. The white asterisk plots the average literature helix angle configuration ($\bar{\boldsymbol{\tau}}\boldsymbol{\cong1}\boldsymbol{0}^{\boldsymbol{o}}\boldsymbol{,}\boldsymbol{\tau}_{\boldsymbol{diff}}\boldsymbol{\cong}\boldsymbol{123}^{\boldsymbol{o}}$).


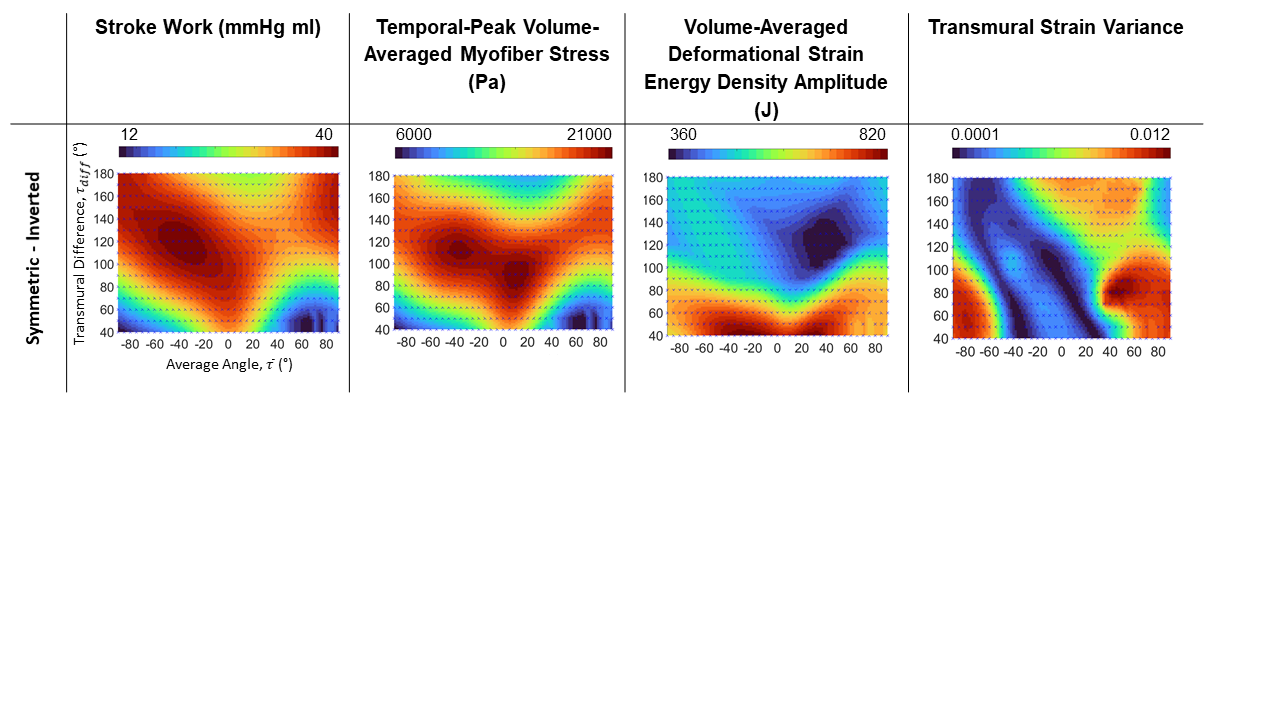


**Figure S5.** Simulation results for fetal “Symmetric” idealised LV model with endo-to-epi inverted helix angles, showing the same characteristic trends as the fetal “Symmetric” idealised model included in Fig 7b. Values on all maps were interpolated from points where data was obtained from simulations (indicated by blue crosses on maps) and all contour maps in this figure contain the same axes as the left figure.

References

Johnson P, Maxwell DJ, Tynan MJ, Allan LD (2000) Intracardiac pressures in the human fetus. Heart 84:59–63. https://doi.org/10.1136/heart.84.1.59

Pennati G, Bellotti M, Fumero R (1997) Mathematical modelling of the human foetal cardiovascular system based on Doppler ultrasound data. Medical Engineering and Physics 19:327–335. https://doi.org/10.1016/S1350-4533(97)84634-6

Vendelin M, Bovendeerd PHM, Engelbrecht J, Arts T (2002) Optimizing ventricular fibers: Uniform strain or stress, but not ATP consumption, leads to high efficiency. American Journal of Physiology - Heart and Circulatory Physiology 283:1072–1081. https://doi.org/10.1152/ajpheart.00874.2001

Versmold HT, Kitterman JA, Phibbs RH, et al (1981) Aortic blood pressure during the first 12 hours of life in infants with birth weight 610 to 4,220 grams. Pediatrics 67:607–613
